# Effects of elevated serum urate on cardiometabolic and kidney function markers in a randomised clinical trial of inosine supplementation

**DOI:** 10.1038/s41598-022-17257-1

**Published:** 2022-07-28

**Authors:** Nicola Dalbeth, Borislav Mihov, Angela Stewart, Gregory D. Gamble, Tony R. Merriman, David Mount, Ian R. Reid, Lisa K. Stamp, Anne Horne

**Affiliations:** 1 Department of Medicine, Faculty of Medical and Health Sciences, University of Auckland, 85 Park Rd, Grafton, Auckland, New Zealand; 2 Department of Biochemistry, University of Otago, Dunedin, New Zealand; 3 Division of Clinical Immunology and Rheumatology, University of Alabama at Birmingham, Birmingham, AL USA; 4 Renal Division, Brigham and Women’s Hospital, VA Boston Healthcare System, Harvard Medical School, Boston, MA USA; 5 Department of Medicine, University of Otago Christchurch, Christchurch, New Zealand

**Keywords:** Biomarkers, Nephrology

## Abstract

In observational studies, serum urate positively associates with cardiometabolic and kidney diseases. We analyzed data from a randomised placebo-controlled trial to determine whether moderate hyperuricemia induced by inosine affects cardiometabolic and kidney function markers. One hundred and twenty post-menopausal women were recruited into a 6-month randomised, double-blind, placebo-controlled trial of inosine for bone health. Change from baseline in the following pre-specified endpoints was analyzed: body mass index; blood pressure; lipid profile; C-reactive protein; fasting glucose; insulin; HbA1c; serum creatinine; and estimated glomerular filtration rate (eGFR). Despite increases in serum urate levels (+ 0.17 mmol/L at week 6, P < 0.0001), no significant between-group differences were observed in cardiometabolic markers, with the exception of lower fasting glucose concentrations with inosine at week 19. In the inosine group, change in serum urate correlated with change in serum creatinine (r = 0.41, P = 0.0012). However, there was no between-group difference in serum creatinine values. Over the entire study period, there was no significant difference in eGFR (ANCOVA P = 0.13). Reduction in eGFR was greater in the inosine group at Week 13 (mean difference − 4.6 mL/min/1.73 m^2^, false detection rate P = 0.025), with no between-group difference in eGFR at other time points. These data indicate that increased serum urate does not negatively influence body mass index, blood pressure, lipid profile, or glycaemic control. Serum urate changes associated with inosine intake correlate with changes in serum creatinine, but this does not lead to clinically important reduction in kidney function over 6 months.

**Clinical trial registration number:** Australia and New Zealand Clinical Trials Registry (ACTRN12617000940370), registered 30/06/2017.

## Introduction

Elevated serum urate is the major causal risk factor for gout^1^. Elevated serum urate has also been associated with metabolic syndrome, cardiovascular disease, and chronic kidney disease in observational studies^2^. However, it is controversial whether urate has a direct or causal influence on these associated conditions, with Mendelian randomization studies not providing consistent evidence to support a causal role of urate^3^.

Inosine is a purine nucleoside that, when ingested orally, increases extra-renal urate production and, thus, serum urate concentrations. In short-term studies, oral administration of inosine provides a useful experimental technique to study the mechanisms of purine metabolism and urate clearance^4,5^. In addition, oral supplementation of inosine has been used in clinical trials for neuroprotection in multiple sclerosis and Parkinson’s disease^6–9^, due to its ability to increase serum urate concentrations.

Our group has recently reported the results of a 6-month randomised placebo-controlled trial to determine whether moderate hyperuricaemia induced by inosine supplements influences the bone turnover markers procollagen type-I N-terminal propeptide (PINP) and β-C-terminal telopeptide of type I collagen (β-CTX) in post-menopausal women^10^. In this trial, although inosine supplementation led to sustained increases in serum urate over the 6-month study period, it did not alter the bone turnover markers in post-menopausal women.

In view of the well documented associations of elevated serum urate with metabolic, cardiovascular and kidney disease, the trial protocol pre-specified cardiometabolic and kidney function markers as secondary endpoints, for the purposes of safety monitoring. Herein, we present this analysis, which provides an opportunity to examine whether elevated urate has a direct influence on these markers.

## Methods

The study methods have been reported in full^10^. This was a six month randomised, double-blind, placebo-controlled trial of 120 post-menopausal female participants, undertaken at a single study site (Clinical Research Centre, University of Auckland) in Auckland, New Zealand. The study was approved by the New Zealand Ministry of Health Southern Health and Disability Ethics Committee (17/STH/102), and all participants provided written informed consent. The trial was prospectively registered with the Australia and New Zealand Clinical Trials Registry (ACTRN12617000940370, registered 30/06/2017). Participants had a screening visit within a month of the baseline study visit, a study visit with randomisation at 0 weeks, and further study visits at 6 weeks, 13 weeks, 19 weeks, and 26 weeks.

### Recruitment and inclusion criteria

As previously reported^10^, letters of invitation were sent to women aged 55 years and over, randomly selected from the New Zealand parliamentary electoral roll. Inclusion criteria were: Age > 55 years; post-menopausal; female; estimated glomerular filtration rate (eGFR) > 60 mL/min/1.73 m^2^; serum urate < 0.42 mmol/L (7 mg/dL); able to provide written informed consent and attend study visits. Exclusion criteria were bone mineral density (BMD) T score < − 2.5 at the total hip, femoral neck or lumbar spine; previous fragility fracture of the hip or clinical vertebral fracture; current or past use within 12 months of medications that can affect bone turnover markers including bisphosphonates and hormone replacement therapy, or any past zoledronate use; history of gout; history of kidney stones; history of diabetes mellitus; current use of diuretic medications; urine pH ≤ 5.0 (risk factor for uric acid urolithiasis); and current use of inosine as a nutritional supplement.

### Interventions

As previously reported^10^, participants were randomised to one of two groups; either placebo or inosine (60 participants per group). Treatment assignment was allocated randomly within blocks of varying size using random numbers drawn from a pseudorandom number generator (Excel 2010)^11^.

Inosine and placebo tablets were the same colour and had identical packaging. Placebo tablets consisted of lactose, microcrystalline cellulose, and magnesium stearate. Inosine tablets contained 500 mg inosine and were compounded by Optimus Healthcare, a registered pharmacy that specialises in compounding for pharmaceutical formulations. Participants were asked to take study medication in the morning and the evening (before 7 pm). All participants were advised to drink 2 L fluid per day.

Pre-labelling of bottles for tablets was performed by staff members who had no contact with the study participants, and no role in study procedures such as assessments of end-points. All personnel having contact with study participants and the participants themselves were blinded to treatment allocation and serum urate concentrations during the study. Serum urate was checked at each study visit, using the Roche Cobas 8000 series automated platform. If the serum urate was measured as 0.48 mmol/L (8 mg/dL) or higher, the inosine dose was reduced by 500 mg. To maintain blinding for study participants and staff, serum urate measurements were visible only to staff members who had no contact with study participants. Each participant in the inosine group was matched by the study statistician to a participant in the placebo group who had placebo dose reduction at the same study visit in the event of dose reduction in the participant in the inosine group.

### Study endpoints

Change from baseline at six-months in the following measures were pre-specified secondary endpoints (measured at each study visit): body mass index; blood pressure (systolic and diastolic); lipid profile (LDL, HDL, triglycerides); C-reactive protein; fasting glucose; and fractional excretion of uric acid (FEUA); serum creatinine; Change from baseline in the following measures were pre-specified exploratory endpoints (measured at baseline and week 26): insulin and HbA1c. Waist circumference, urinary uric acid/urinary creatinine ratio (UUA/Ucr ratio), and eGFR were also measured at each study visit, and for the purposes of completeness, are also reported in this paper.

### Assessment procedures

As previously reported^10^, height was measured at the baseline visit using a Harpenden stadiometer. Weight was measured at each study visit using electronic scales, and body mass index (BMI) was calculated in kg/m^2^. Blood pressure was measured using a Dinamap automatic monitor at baseline, with a specified arm position, sphygmomanometer cuff application, and a 5 min period of rest before measurement. The device automatically takes three measurements over a three minute period, during which time the participant remains undisturbed. The mean of the second and third measurements was used for analysis of systolic and diastolic blood pressure.

Fasting blood samples were collected at each study visit, and tested for urate (blinded, as described above), creatinine, lipid profile, glucose, and C-reactive protein, using the cobas 8000 modular analyzer series (Roche). HbA1c and insulin were tested at baseline and at 26 weeks. HbA1c was measured using the Capillarys 2 analyzer (Sebia), and insulin was measured on the cobas e 411 analyzer (Roche).

eGFR was calculated using the Modification of Diet in Renal Disease (MDRD) formula^12^.

FEUA was calculated as the clearance of uric acid/clearance of creatinine, expressed as a percentage. UUA/Ucr ratio calculated as urinary uric acid concentation/urinary creatinine concentation.

Adverse events and serious adverse events were recorded at all follow-up visits. Adverse effects were monitored by a blinded independent safety monitor.

### Sample size calculation

As previously reported, the sample size was calculated based on the primary endpoint of bone turnover markers (βCTX and P1NP)^10^. This was a pre-planned secondary safety analysis of data. The study size was limited to the inception cohort.

### Statistical analysis

Data are presented as mean (standard deviation (SD)) or n (%) for descriptive purposes; however, measures of effect are presented with the appropriate 95% confidence interval. Data were analysed on an intention to treat basis, using a mixed models approach to repeated measures. For analysis of change from baseline, a mixed-models analysis of covariance (ANCOVA) approach to repeated measures was used. For ANCOVA, the dependent variable was change from baseline, and baseline level was included as a covariate in the analysis. False detection rate protected pairwise comparisons were performed at each time point using the mixed model variances. Pearson’s correlations were used to determine relationships between change in serum urate and change in other variables over the study periods, as well as change in FEUA and UUA/UCr ratio and kidney function measures. For the correlation analysis, the change over the study period was calcuated using the average change over Weeks 6, 13, 19 and 26 for each variable. All analyses were performed using SAS (SAS Institute Inc v 9.4, Cary, NC, USA).

### Ethics approval and consent to participate

This study complied with the Declaration of Helsinki, the New Zealand Ministry of Health Southern Health and Disability Ethics Committee (17/STH/102) approved the research protocol, and written informed consent was obtained from all study participants.

## Results

### Baseline characteristics

Participant characteristics at the baseline visit, including cardiometabolic and kidney function markers are shown in Table 1. The mean baseline serum urate was 0.28 mmol/L (4.7 mg/dL) in the placebo group, and 0.27 mmol/L (4.5 mg/dL) in the inosine group. FEUA was 8% in both groups. Except for waist circumference which was higher in the placebo group (mean 89 cm compared with 85 cm in the inosine group), physical measurements and tested laboratory markers were similar between the two groups at baseline.

**Table 1:** Characteristics of study participants at baseline.

	Placebo (n = 60)	Inosine (n = 60)
Age (years)	68.8 (1.9)	68.2 (2.3)
**Ethnicity, n (%)**		
NZ European	59 (98%)	58 (97%)
NZ Māori	1 (2%)	0
NZ Asian	0	2 (3%)
**Serum urate**		
mmol/L	0.28 (0.06)	0.27 (0.06)
mg/dL	4.7 (1.0)	4.5 (1.0)
Weight (kg)	72.4 (12.6)	67.5 (10.9)
Body mass index (kg/m^2^)	27.3 (4.7)	26.1 (4.2)
Waist circumference (cm)	89 (12)	85 (11)
Systolic blood pressure (mmHg)	136 (17)	134 (18)
Diastolic blood pressure (mmHg)	73 (8)	72 (10)
LDL cholesterol (mmol/L)	3.0 (0.9)	3.2 (0.8)
HDL cholesterol (mmol/L)	1.8 (0.4)	1.9 (0.5)
Triglycerides (mmol/L)	1.0 (0.4)	1.0 (0.4)
C-reactive protein (mg/L)	2.3 (2.5)	2.0 (1.9)
Fasting glucose (mmol/L)	5.0 (0.5)	4.9 (0.4)
HbA1c (mmol/mol)	37 (3)	36 (3)
Insulin (mU/L)	8.7 (4.1)	7.8 (3.4)
FEUA (%)	8.0 (3.1)	8.0 (2.7)
Creatinine (µmol/L)	63.3 (8.6)	62.5 (8.7)
eGFR (mL/min/1.73 m^2^)	91 (15)	92 (14)

Unless otherwise stated, data are presented as mean (SD). Baseline demographic values, weight, body mass index, serum creatinine, and serum urate have been reported previously^10^.

### Serum urate

**Figure 1:**
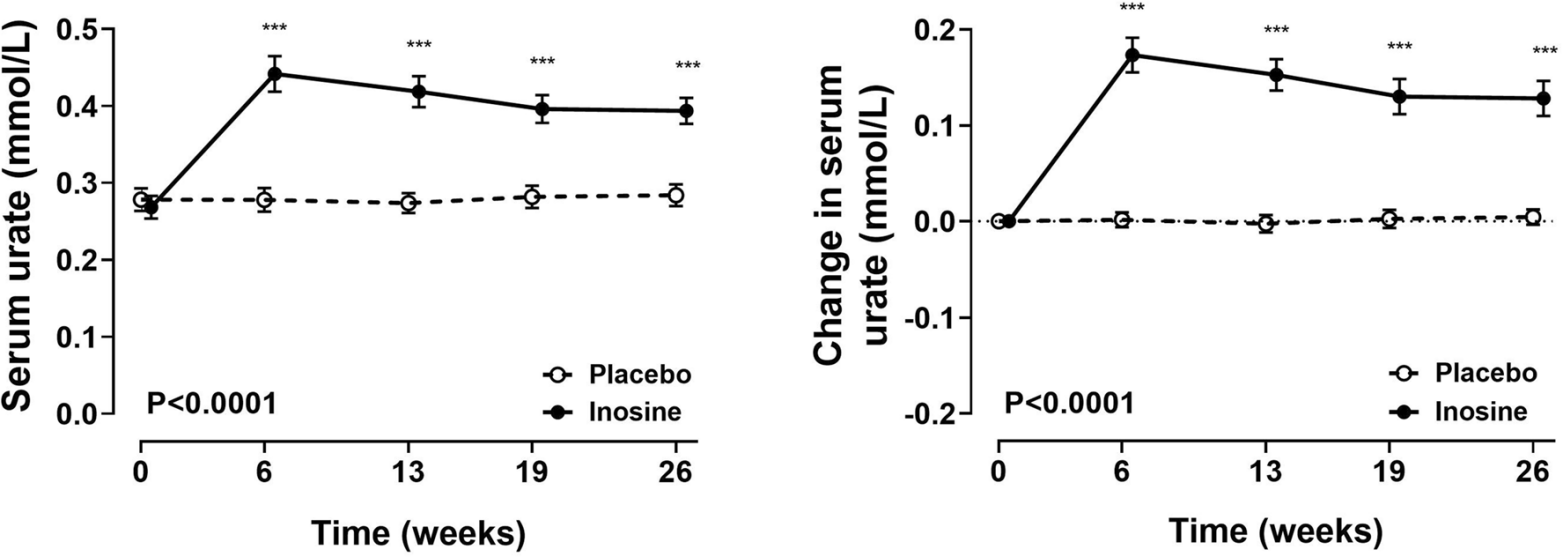
Serum urate. Absolute values (left panel) and change from baseline (right panel). Absolute serum urate values have been reported previously^10^. Data are shown as mean (95% CI), with P_time×treatment_. False discovery rate ***P < 0.001.

As previously reported^10^, administration of inosine supplements led to a significant increase in serum urate, which was maintained over the study period (ANCOVA P < 0.0001, P for all follow-up time-points < 0.0001; Fig. 1). In the inosine group, serum urate levels increased from baseline by + 0.17 mmol/L (+ 2.9 mg/dL) at week 6, and remained increased throughout the study period; by + 0.13 mmol/L (+ 2.2 mg/dL) at week 26.

### Physical measurements and blood pressure

**Figure 2:**
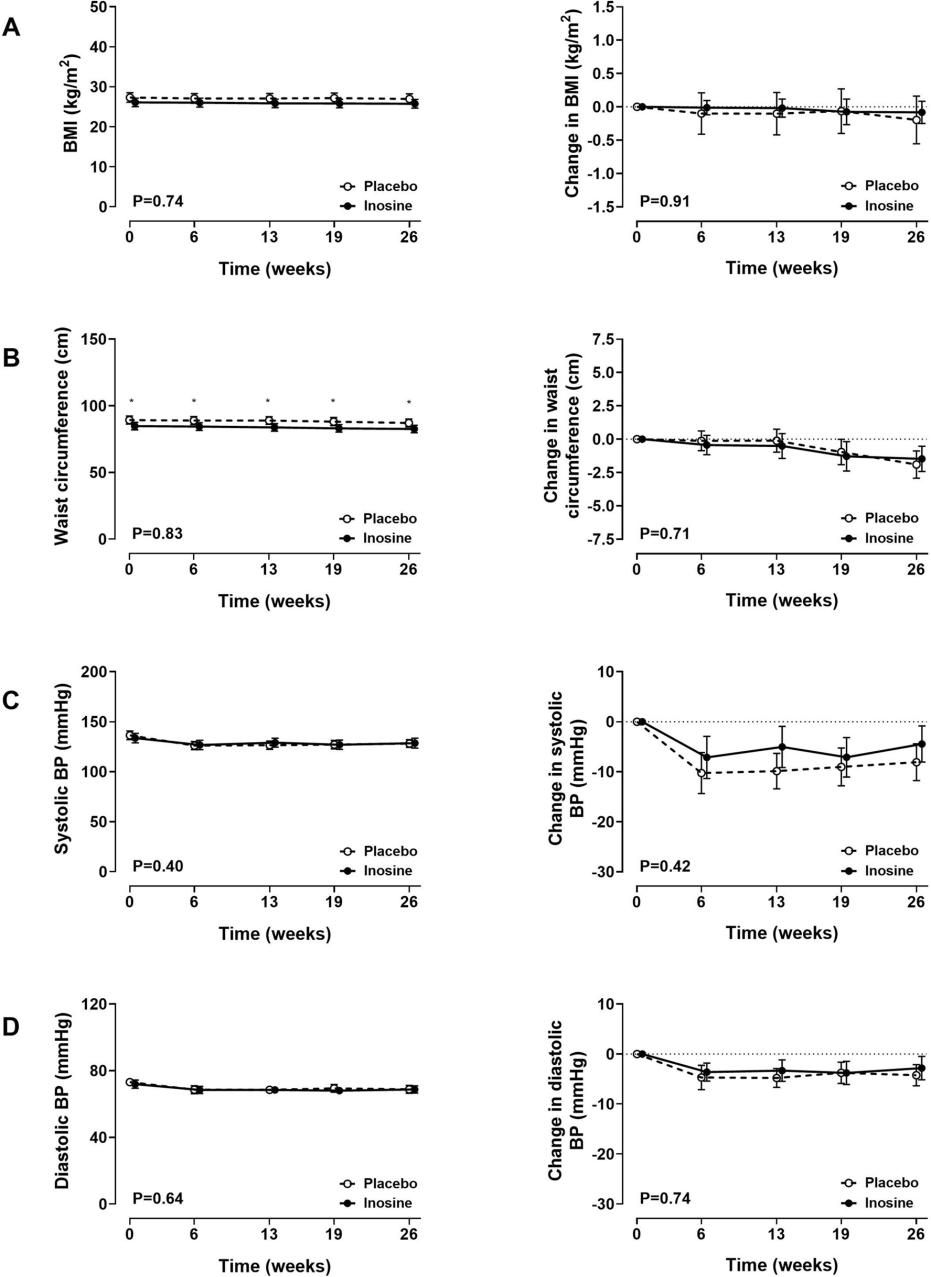
Physical measurements and blood pressure. Absolute values (left panel) and change from baseline (right panel). (**A**) Body mass index; (**B**) waist circumference; (**C**) systolic blood pressure; (**D**) diastolic blood pressure. Data are shown as mean (95% CI), with P_time×treatment_. False discovery rate *P < 0.05.

Body mass index (BMI) did not change in either group, and there were no between-group differences in BMI at any time point (ANCOVA P** = **0.91; Fig. 2A). Waist circumference was higher at baseline in the placebo group, and remained so throughout the study. In both groups, there was a small reduction in waist circumference at week 26, with no between-group difference in the change in waist circumference (ANCOVA P** = **0.71; Fig. 2B). Both systolic and diastolic blood pressure reduced from baseline at all study visits from week 6, and there were no between-group differences in blood pressure (ANCOVA P > 0.41; Fig. 2C,D). There was no significant correlation between change in serum urate over the study period, and change in BMI or waist circumference (Supplementary Table 1). In the inosine group, there was a negative correlation between change in serum urate and change in diastolic blood pressure (r =  − 0.26, P = 0.048), with a similar trend for change in systolic blood pressure (r =  − 0.26, P = 0.051, Supplementary Table 1).

### Lipid profile and C-reactive protein

**Figure 3:**
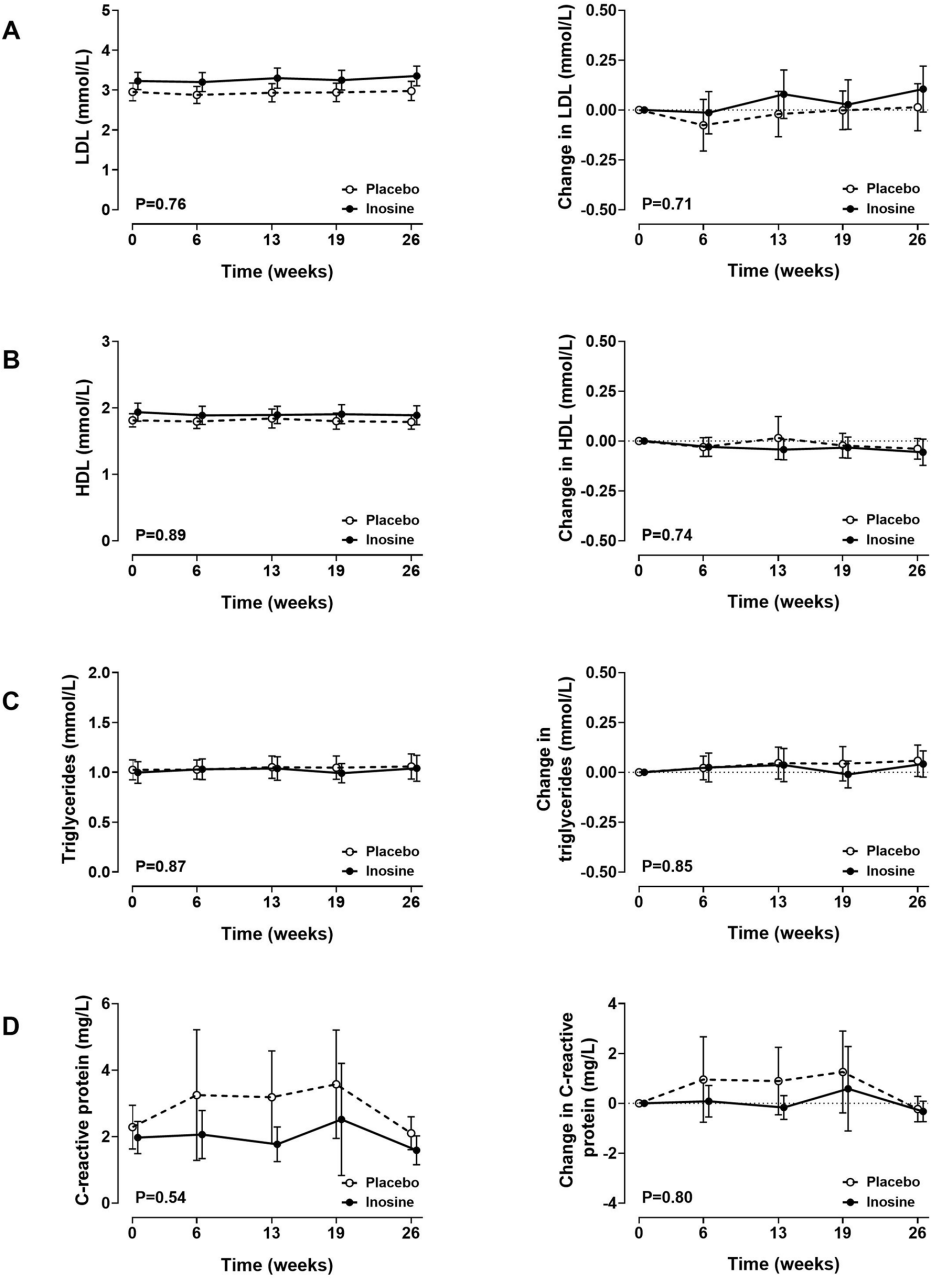
Lipid profile and C-reactive protein. Absolute values (left panel) and change from baseline (right panel). (**A**) LDL cholesterol; (**B**) HDL cholesterol; (**C**) triglycerides; (**D**) C-reactive protein. Data are shown as mean (95% CI), with P_time×treatment_.

LDL cholesterol, HDL cholesterol, and triglycerides remained unchanged throughout the study, with no between-group differences observed at any time point (ANCOVA P** > **0.70; Fig. 3A–C). Similarly, there was no statistically significant change in C-reactive protein in either group, and no between-group difference in C-reactive protein (ANCOVA P = 0.80; Fig. 3D). There was no significant correlation between change in serum urate over the study period, and change in lipid profile or C-reactive protein (Supplementary Table 1).

### Glycaemic control and circulating insulin levels

**Figure 4:**
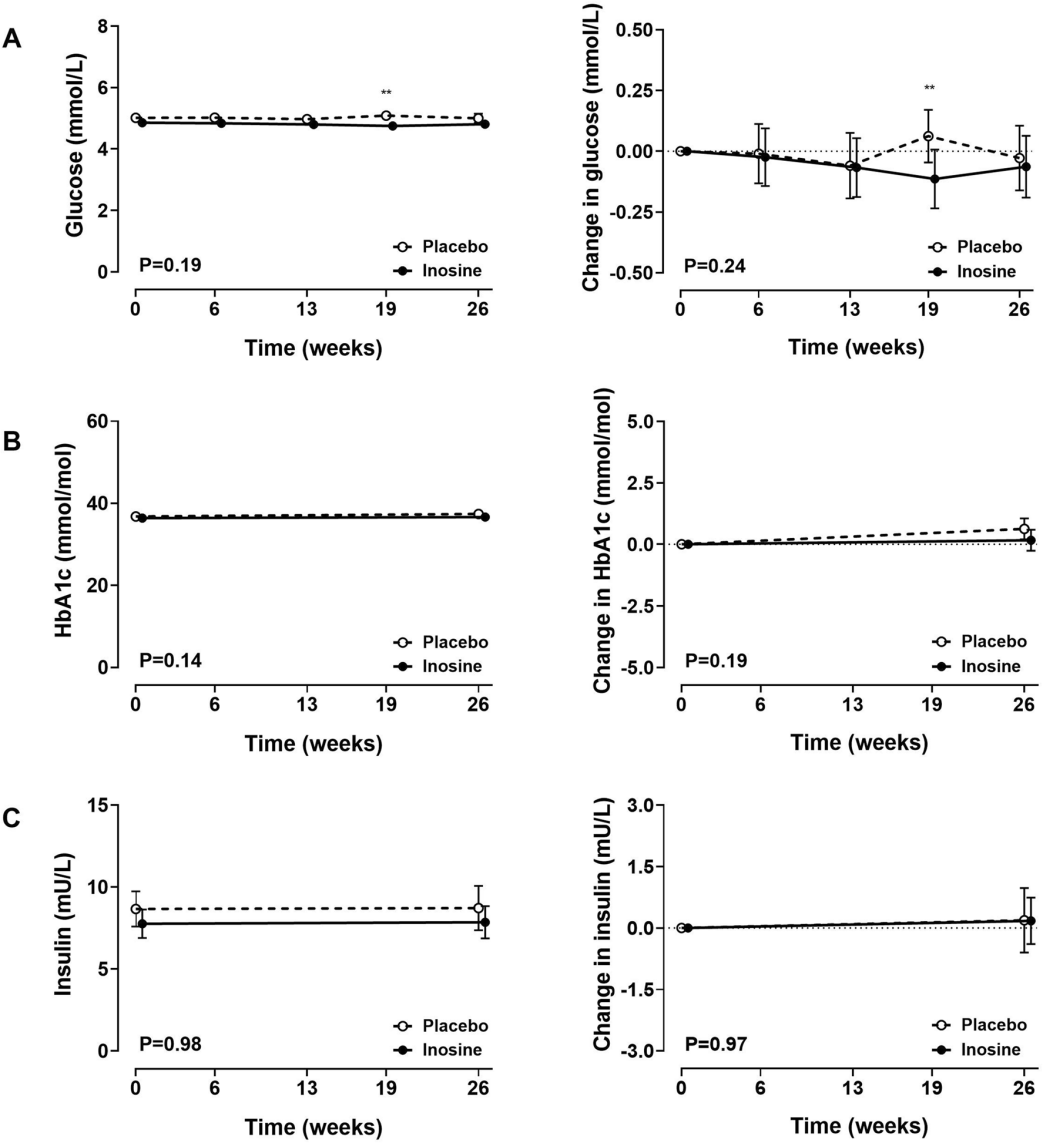
Glycaemic control and circulating insulin levels. Absolute values (left panel) and change from baseline (right panel). (**A**) Fasting glucose; (**B**) HbA1c; (**C**) insulin. Data are shown as mean (95% CI), with P_time×treatment_. False discovery rate **P < 0.01.

Over the entire study period, there were no statistically significant differences in fasting glucose concentrations, HbA1c, or insulin (ANCOVA P > 0.18; Fig. 4). At week 19, fasting glucose concentrations were lower in the inosine group, compared with the placebo group, but this difference was not observed at other time points (Fig. 4A). There were no between-group differences in HbA1c or insulin (Fig. 4B,C). There was no significant correlation between change in serum urate over the study period, and change in markers of glycaemic control (Supplementary Table 1).

### FEUA, UUA/UCr ratio, and measures of kidney function

**Figure 5:**
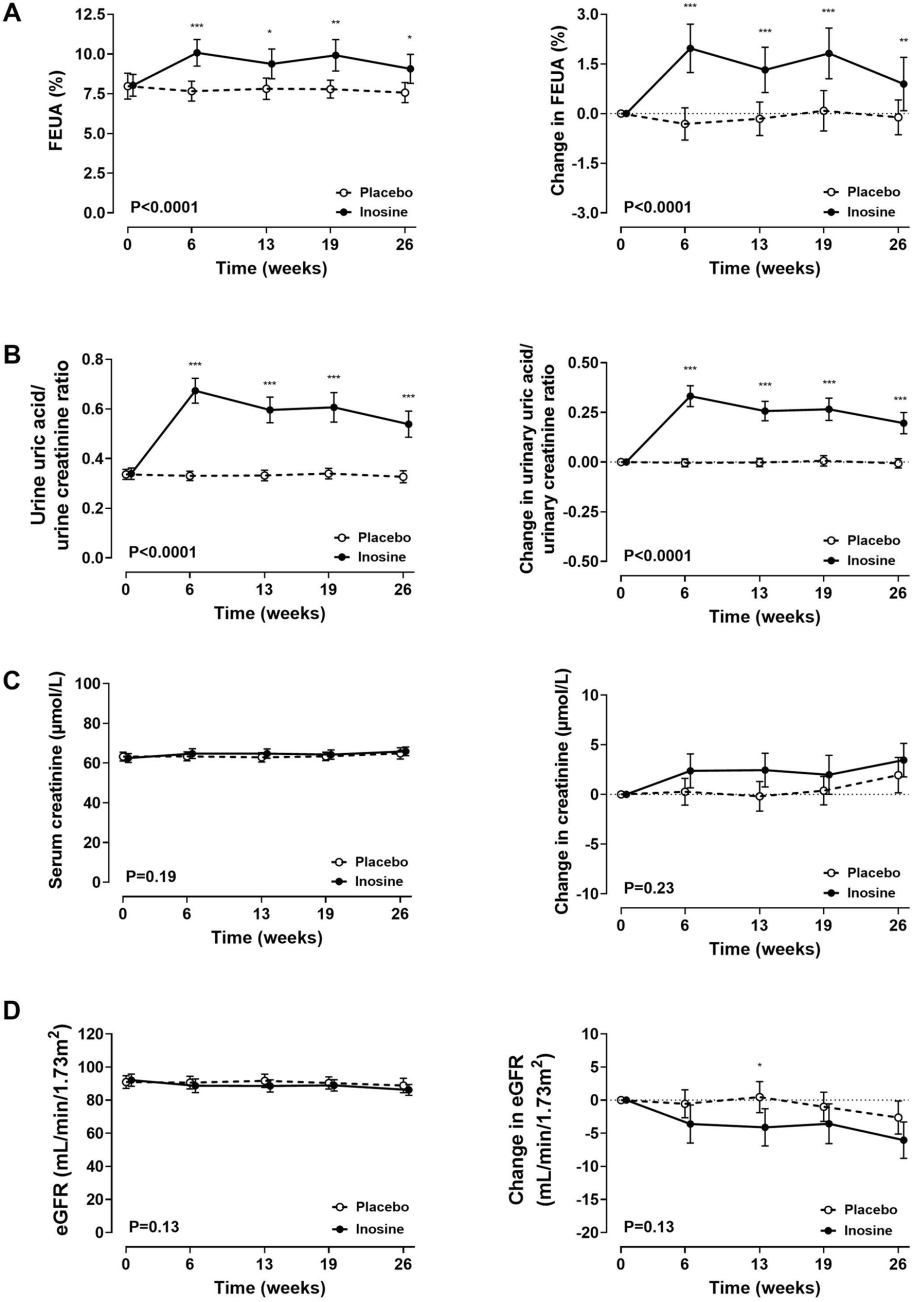
Fractional excretion of uric acid (FEUA), urinary uric acid/urinary creatinine ratio, and measures of kidney function. Absolute values (left panel) and change from baseline (right panel). (**A**) FEUA; (**B**) Urinary uric acid/urinary creatinine ratio. (**C**) Serum creatinine; (**D**) eGFR. Data are shown as mean (95% CI), with P_time×treatment_. False discovery rate ***P < 0.001, **P < 0.01, *P < 0.05.

Administration of inosine led to a statistically significant increase in FEUA over the study period (ANCOVA P < 0.0001, P for all follow-up time-points < 0.05; Fig. 5A). The maximum difference between groups in FEUA was observed at week 6; at this time point, the mean (SD) FEUA was 10.1 (3.2)% in the inosine group and 7.7 (2.4)% in the placebo group (P < 0.0001). Similarly, administration of inosine led to a statistically significant increase in the UUA/UCr ratio over the study period (ANCOVA P < 0.0001, P for all follow-up time-points < 0.001; Fig. 5B).

There was a small increase in serum creatinine in both groups over the study period, but no between-group difference in serum creatinine values at any time point over the 6-month period (ANCOVA P = 0.23; Fig. 5C). Analysis of the change in serum creatinine from baseline showed a maximal difference between groups at week 13 (with mean (95% CI) difference of 2.6 (95% CI 0.4, 4.9) µmol/L, false detection rate P = 0.059). At all other time points, there was no between-group difference in the change in serum creatinine.

Consistent with the serum creatinine results, there was a small decline in eGFR over the 6-month study period in both groups. Over the entire study period, there was no significant difference in eGFR (ANCOVA P = 0.13; Fig. 5D). However, reduction in eGFR was greater in the inosine group at week 13 (mean difference − 4.6 (95% CI − 8.2, − 1.0) mL/min/1.73m^2^, false detection rate P = 0.025), with no between-group difference in eGFR at other time points.

In correlation analysis of all study participants, change in serum urate positively correlated with change in serum creatinine (r = 0.32, P = 0.0004), and negatively correlated with change in eGFR (r =  − 0.33, P = 0.0003, Supplementary Table 1). Analysis of the participants in the placebo group showed no correlation for change in serum urate with change in serum creatinine or change in eGFR in the placebo group (Supplementary Table 1). However, for participants in the inosine group, change in serum urate positively correlated with change in serum creatinine (r = 0.41, P = 0.0012), and negatively correlated with change in eGFR (r =  − 0.37, P = 0.004, Supplementary Table 1). However, neither change in FEUA nor change in UUA/UCr ratio correlated with change in serum creatinine or with change in eGFR (Supplementary Tables 2, 3).

### Cardiovascular, metabolic, and kidney adverse events

Two cardiovascular adverse events were reported during the study; a new diagnosis of hypertension in one participant in the placebo group and in one participant in the inosine group. One participant in the inosine group experienced a serious adverse event of ischaemic colitis. No participant had a new diagnoses of diabetes mellitus. No episodes of acute kidney injury or urolithiasis were observed.

## Discussion

This clinical trial of inosine supplementation provided an important opportunity to examine whether elevated serum urate has a direct influence on markers of metabolic, cardiovascular and kidney disease over a 6-month period. Despite large increases in serum urate in the inosine group, no significant between-group differences were observed over the study period in body mass index, blood pressure, lipid profile, C-reactive protein, or markers of glycaemic control, with the exception of lower fasting glucose concentrations with inosine at a single time point. These data do not provide support for the hypothesis that elevated serum urate contributes directly to elevated body mass index, hypertension, dyslipidaemia, or diabetes mellitus. Overall, there were no between-group differences in serum creatinine, although there was a greater reduction in eGFR at a single time point with inosine. The findings indicate that elevated serum urate due to increased oral purine load does not lead to kidney impairment over a 6 month period.

Despite large increases in serum urate in the inosine group, there were no statistically significant differences in serum creatinine between the inosine and placebo groups. These findings are broadly consistent with two recent randomized placebo-controlled clinical trials of the urate-lowering medication allopurinol, which did not demonstrate that allopurinol prevented progression of chronic kidney disease^13,14^. Administration of inosine provides a useful model to study effects of serum urate elevations independent of kidney function, as tubulointerstitial disease may lead to elevated serum urate due to renal under-excretion and is also an important risk factor for chronic kidney disease progression^15^. The fractional excretion of urate increased after inosine loading; increased urate excretion in the setting of uricosuric therapy and genetic deficiencies in URAT1 or GLUT9 has also been implicated in the genesis of acute kidney injury and/or nephrolithiasis, likely due to the uricosuria with intra-tubular crystallization of monosodium urate^16–18^. It is important to recognise that hyperuricaemia in non-experimental settings is mostly due to renal under-excretion of urate (due to reduced kidney function and genetic variants in renal urate transporters that collectively lead to increased reabsorption of urate)^19,20^, and that the effects on the kidneys of elevations of serum urate due to over-production (due to an oral purine load) may differ to elevated urate induced by renal under-excretion. Oral administration of inosine may also lead to non-urate mediated effects of other purine metabolites. Longer exposure to elevated serum urate beyond the six-month duration of this study could also have a cumulative effect on kidney function.

The study findings in post-menopausal women align with results from the recent SURE-PD3 trial of inosine for early Parkinson Disease progression^9^. In SURE-PD3, 298 people with early Parkinson Disease were randomised to placebo or inosine dosed by blinded titration to increase serum urate concentrations to 0.42 -0.48 mmol/L (7.1–8.0 mg/dL) for up to 2 years. In the inosine group, the mean serum urate increased from 0.27 mmol/L (4.6 mg/dL) to around 0.42 mmol/L (7 mg/dL) over the study period. No difference was observed in the primary outcome measure of Parkinson Disease clinical progression rate, and the trial was stopped early following a pre-specified interim futility analysis. In addition, there were no between-group differences in body mass index, systolic or diastolic blood pressure, fasting glucose, or lipid profile over the 2-year study period. There was greater decline in eGFR in the inosine group (mean difference between groups − 3.71 mL/min/1.73m^2^, P < 0.0001). Higher rates of kidney stones were also observed in the inosine group (rate ratio 4.92). In contrast to SURE-PD3, our study of post-menopausal women did not demonstrate significant between-group differences in eGFR or higher risk of kidney stones. The larger sample size and longer duration of SURE-PD3 may account for these differences, but the different study populations (51% men in SURE-PD3) may also have contributed.

Study strengths include the comprehensive pre-specified assessment of cardiometabolic and kidney function markers in a double-blind randomised controlled trial. The study also has limitations. Although there was no observed difference in cardiovascular, metabolic, or kidney adverse events, the sample size and study duration limited definitive conclusions about long-term safety of inosine. All participants had normal kidney function at baseline, normal urine pH, and no history of kidney stones. The study results may not be generalizable to those with chronic kidney disease or prior kidney stones. Urine markers of kidney function, including urine albumin:creatinine ratio were not analysed as part of the study. Additionally, the study results may not be generalizable to non-European populations, men, or younger people.

## Conclusions

Inosine supplementation leads to a large increase in serum urate, but does not negatively impact on body mass index, blood pressure, lipid profile, C-reactive protein, or markers of glycaemic control. In addition, serum urate changes associated with inosine use correlate with changes in serum creatinine, but this does not lead to clinically important reduction in kidney function over 6 months.

## Supplementary Information

Supplementary Tables.

## Data Availability

The data underlying this article cannot be shared publicly. The data will be shared on reasonable request to the corresponding author.

## Abbreviations

ANCOVA: Analysis of covariance
βCTX: C-terminal cross-linked telopeptide of type I collagen
BMD: Bone mineral density
BMI: Body mass index
eGFR: Estimated glomerular filtration rate
FEUA: Fractional excretion of uric acid
GLUT9: Solute carrier family 2, facilitated glucose transporter member 9
HbA1c: Glycated hemoglobin
HDL: High-density lipoprotein
LDL: Low-density lipoprotein
P1NP: Procollagen-1N-terminal peptide
SD: Standard deviation
SURE-PD3: Study of urate elevation in Parkinson’s disease, phase 3
URAT1: Urate transporter 1
UUA/Ucr ratio: Urinary uric acid/urinary creatinine ratio
