# Supplementary material for: Effects of elevated serum urate on cardiometabolic and kidney function markers in a randomised clinical trial of inosine supplementation
Source: Sci Rep. 2022 Jul 28;12:12887. doi: 10.1038/s41598-022-17257-1 (PMC9334273; doi:10.1038/s41598-022-17257-1)
Supplement: Supplementary file 1 — Supplementary Tables. [file 41598_2022_17257_MOESM1_ESM.docx]

**Supplementary Table 1.** Pearson correlation analysis between net change in serum urate over the study period and net change in mean circulating markers over the study period. Net change was calculated as the (average of Weeks 6, 13, 19, 26 weeks value)- baseline value.

| **Variable** |  | **All** | **Placebo** | **Inosine** |
| --- | --- | --- | --- | --- |
| Body mass index (kg/m^2^) | r  P | 0.05  0.58 | 0.17  0.20 | -0.05  0.69 |
| Waist circumference (cm) | r  P | -0.05  0.56 | -0.11  0.40 | -0.06  0.66 |
| Systolic blood pressure (mmHg) | r  P | 0.01  0.89 | -0.26  0.05 | -0.26  0.05 |
| Diastolic blood pressure (mmHg) | r  P | -0.04  0.67 | -0.13  0.32 | -0.26  0.048 |
| LDL cholesterol (mmol/L) | r  P | 0.13  0.17 | -0.17  0.20 | 0.22  0.09 |
| HDL cholesterol (mmol/L) | r  P | -0.02  0.80 | -0.15  0.27 | 0.15  0.25 |
| Triglycerides (mmol/L) | r  P | -0.0  0.37 | 0.02  0.89 | 0.16  0.24 |
| C-reactive protein (mg/L) | r  P | -0.18  0.05 | -0.27  0.04 | -0.09  0.50 |
| Fasting glucose (mmol/L) | r  P | -0.09  0.33 | 0.00  0.97 | -0.09  0.50 |
| HbA1c (mmol/mol) | r  P | -0.12  0.22 | -0.12  0.38 | 0.10  0.47 |
| Insulin (mU/L) | r  P | -0.01  0.93 | 0.16  0.22 | 0.14  0.31 |
| Creatinine (µmol/L) | r  P | 0.32  0.0004 | 0.04  0.78 | 0.41  0.0012 |
| eGFR (mL/min/1.73m^2^) | r  P | -0.33  0.0003 | 0.09  0.48 | -0.37  0.004 |

**Supplementary Table 2.** Pearson correlation analysis between net change in FEUA over the study period and net change in average kidney function measures over the study period. Net change was calculated as the (average of Weeks 6, 13, 19, 26 weeks value) - baseline value.

| **Variable** |  | **All** | **Placebo** | **Inosine** |
| --- | --- | --- | --- | --- |
| Creatinine (µmol/L) | r  P | 0.11  0.25 | 0.18  0.17 | -0.07  0.60 |
| eGFR (mL/min/1.73m^2^) | r  P | -0.13  0.15 | -0.23  0.08 | 0.05  0.72 |

**Supplementary Table 3.** Pearson correlation analysis between net change in urinary uric acid/urinary creatinine over the study period and net change in average kidney function measures over the study period. Net change was calculated as the (average of Weeks 6, 13, 19, 26 weeks value)- baseline value.

| **Variable** |  | **All** | **Placebo** | **Inosine** |
| --- | --- | --- | --- | --- |
| Creatinine (µmol/L) | r  P | 0.05  0.57 | -0.18  0.17 | -0.17  0.21 |
| eGFR (mL/min/1.73m^2^) | r  P | -0.08  0.40 | 0.11  0.42 | 0.15  0.24 |
